# Downregulation of TNFAIP3 Associated With Poor Prognosis and Immune Response in Breast Cancer

**DOI:** 10.1002/cnr2.70443

**Published:** 2025-12-29

**Authors:** Yeqin Wu, Zhaoyang Qin, Gangping Wang

**Affiliations:** ^1^ Department of Pathology The Fourth Affiliated Hospital of School of Medicine, & International School of Medicine, International Institutes of Medicine, Zhejiang University Zhejiang China; ^2^ Department of General Surgery The Rizhao People's Hospital Rizhao China; ^3^ Central Laboratory, the Rizhao People's Hospital Rizhao China

**Keywords:** breast cancer (BRCA), immune infiltration, LINC01096, prognostic biomarker, TNFAIP3, tumor microenvironment

## Abstract

**Background:**

Breast cancer (BRCA) progression is closely linked to dysregulated inflammatory processes within the tumor microenvironment (TME). TNFAIP3, a key regulator of TNF‐α signaling, plays a dual role in cancer biology, but its precise function in BRCA pathogenesis and immune modulation remains unclear.

**Aims:**

This study aimed to elucidate the clinical significance, immune regulatory role, and molecular mechanisms of TNFAIP3 in BRCA progression.

**Methods and Results:**

We conducted an analysis of RNA‐seq data from 1113 BRCA samples and 113 normal samples sourced from TCGA, along with protein data from 125 BRCA samples and 18 normal samples obtained from the CPTAC database. Comprehensive analyses included: (1) differential expression across cancer types and BRCA subtypes; (2) correlation with clinicopathological features; (3) survival analysis using Kaplan–Meier and Cox regression; (4) immune cell infiltration assessment via ssGSEA; and (5) co‐expression analysis of TNFAIP3‐LINC01096. TNFAIP3 was significantly downregulated in BRCA (*p* < 0.001) and associated with aggressive features, including advanced TNM stage (T4 vs. T1, *p* = 0.021) and poor prognosis (HR = 1.48, *p* = 0.035). Immune profiling revealed strong correlations with cytotoxic T cells (*ρ* = 0.632) and dendritic cells (*ρ* = 0.602). A novel TNFAIP3‐LINC01096 regulatory network was identified (*ρ* = 0.582, *p* < 0.001), potentially mediated by shared miR‐3130‐3p binding sites.

**Conclusion:**

Our findings suggest a role of TNFAIP3 as a critical regulator of BRCA progression and tumor immunity. The TNFAIP3‐LINC01096 axis represents a promising therapeutic target, particularly for immunotherapy‐resistant cases. These results provide a rationale for developing TNFAIP3‐based prognostic tools and immunomodulatory strategies in BRCA management.

**Abbreviations**
ACC: adrenocortical carcinoma
aDC: activated dendritic cells
APCs: antigen‐presenting cells
AUC: the area under the curve
BLCA: bladder urothelial carcinoma
BRCA: breast cancer
CCLs: C‐C motif chemokine ligands
CESC: cervical squamous cell carcinoma and endocervical adenocarcinoma
CHOL: Cholangio carcinoma
COAD: colon adenocarcinoma
CXCLs: C‐X‐C motif chemokine ligands
DC: dendritic cells
DLBC: diffuse large B‐cell lymphoma
ER: estrogen receptor
ESCA: esophageal carcinoma
GBM: glioblastoma multiforme
HER2: human epidermal growth factor receptor 2
HNSC: head and neck squamous cell carcinoma
iDC: immature dendritic cells
IDC: infiltrating ductal carcinoma
IFN: interferon
IL: interleukin
ILC: infiltrating lobular carcinoma
JAK–STAT: Janus kinase/signal transducers and activators of transcription
KICH: kidney chromophobe
KIRC: kidney renal clear cell carcinoma
KIRP: kidney renal papillary cell carcinoma
LAML: acute myeloid leukemia
LGG: brain lower grade glioma
LIHC: liver hepatocellular carcinoma
LINC01096: long non‐coding RNA 1096
lncRNA: long non‐coding RNA
LUAD: lung adenocarcinoma
LUSC: lung squamous cell carcinoma
MAPK: mitogen‐activated protein kinase
MESO: mesothelioma
Mixed: mixed histology
NF‐κB: nuclear factor kappa‐B
NK: natural killer cells
NST: invasive breast carcinoma of no special type
OV: ovarian serous cystadenocarcinoma
PAAD: pancreatic adenocarcinoma
PCA: principal component analysis
PCPG: pheochromocytoma and paraganglioma
PCs: principal components
pDC: plasmacytoid dendritic cells
PR: progesterone receptor
PRAD: prostate adenocarcinoma
READ: rectum adenocarcinoma
ROC: receiver operating characteristic
SARC: sarcoma
SKCM: skin cutaneous melanoma
SNPs: single‐nucleotide polymorphisms
STAD: stomach adenocarcinoma
Tcm: T central memory
TFH: T follicular helper
TGCT: testicular germ cell tumors
Tgd: T gamma delta
TGF‐β: transforming growth factor‐β
Th1: T helper1
THCA: thyroid carcinoma
THYM: thymoma
TLR: toll‐like receptor pathways
TNBC: triple‐negative breast cancer
TNFAIP: tumor necrosis factor alpha‐induced protein
TNF‐α: tumor necrosis factor‐alpha
TNM: tumor‐node‐metastasis
UCEC: uterine corpus endometrial carcinoma
UCS: uterine carcinosarcoma
UMAP: uniform manifold approximation and projection
UMIs: unique molecular identifiers
UVM: uveal melanoma

## 1 Introduction

Breast cancer (BRCA) is the most prevalent malignant tumor among women globally and the primary cause of cancer‐related deaths [1, 2, 3, 4]. Despite significant progress in diagnosis and treatment, the molecular mechanisms underlying BRCA progression and immune evasion remain incompletely understood [5]. Emerging evidence highlights the critical role of chronic inflammation and immune dysregulation in BRCA pathogenesis [6, 7, 8]. The tumor microenvironment (TME), characterized by complex interactions between cancer cells and immune components, plays a pivotal role in tumor progression and therapeutic response [9, 10]. In particular, TNF‐α has been implicated as a master regulator of these processes [9].

TNF‐α is a pleiotropic cytokine, functioning as a pivotal mediator in inflammation and immune responses [9, 11, 12, 13, 14]. Under physiological conditions, TNF‐α maintains tissue homeostasis through tightly regulated signaling cascades [15]. However, in cancer, dysregulated TNF‐α signaling contributes to tumor promotion through multiple mechanisms [14, 16, 17, 18]: (1) activation of NF‐κB (nuclear factor kappa‐B) pathway, leading to enhanced cell survival and proliferation [19, 20, 21, 22]; (2) induction of epithelial‐mesenchymal transition (EMT) [23]; and (3) regulation of immune cell function within the TME [24, 25, 26]. The TNF‐α‐induced protein 3 (TNFAIP3), also known as A20, is a key negative feedback regulator of NF‐κB signaling, acting through its dual ubiquitin‐editing functions [27].

The TNFAIP3 protein comprises an N‐terminal ovarian tumor (OTU) domain possessing deubiquitinating enzyme activity, along with seven C‐terminal zinc finger domains exhibiting E3 ubiquitin ligase activity [28, 29, 30]. This unique structure enables TNFAIP3 to modulate inflammatory responses by terminating TNF receptor signaling through: (1) deubiquitination of receptor‐interacting protein (RIP) kinases [31, 32, 33]; (2) inhibition of IκB kinase (IKK) complex activation [33, 34]; and (3) suppression of NF‐κB‐dependent gene expression [35, 36, 37, 38]. In cancer biology, TNFAIP3 has been reported to play context‐dependent roles—acting as either a tumor suppressor by limiting chronic inflammation or as an oncogene by promoting cancer cell survival [39]. This functional duality may stem from tissue‐specific differences in TNFAIP3 regulation and its interaction with various signaling pathways [40, 41, 42].

Our preliminary clinical data research indicates that LINC01096 is upregulated in BRCA and is associated with TNM staging [43]. Our preliminary study also found that miR‐3130‐3p is significantly downregulated in BRCA [43]. Notably, LINC01096 has emerged as a potential regulator of inflammatory responses in BRCA, though its relationship with TNFAIP3 remains unexplored. LINC01096 may function as a competitive endogenous RNA, modulating TNFAIP3 expression via sponging miR‐3130‐3p [43], representing a novel regulatory axis in BRCA immune modulation.

While TNFAIP3's role has been investigated in various malignancies, its expression patterns, prognostic significance, and immune regulatory functions in BRCA remain poorly characterized [27, 39]. Furthermore, the potential crosstalk between TNFAIP3 and immune cell infiltration in the BRCA microenvironment warrants systematic investigation [28, 29, 30, 44]. This study aims to elucidate the clinical significance, immune regulatory role, and explore its potential regulatory relationship with LINC01096 in BRCA. Our findings may provide new insights into BRCA pathogenesis and identify TNFAIP3 as a potential biomarker for immune classification and therapeutic targeting.

## 2 Materials and Methods

### 2.1 Data Acquisition and Preprocessing

We obtained RNA sequencing data (in transcripts per million [TPM] format) and corresponding clinical information for 1113 BRCA samples and 113 normal breast tissue samples from the UCSC Xena database (https://xenabrowser.net/datapages/) [45]. The dataset comprised uniformly processed transcriptomic data from TCGA (The Cancer Genome Atlas) and GTEx (Genotype‐Tissue Expression) projects, which were generated using the Toil pipeline for consistent data processing and normalization [45]. To ensure data homogeneity, we included only female patients who had not received neoadjuvant therapy. Additional clinical annotations were supplemented from Berger et al. [46]. For downstream analyses, gene expression values were log2‐transformed after adding a pseudocount (log2[TPM + 1]) to stabilize variance and improve normality.

For proteomic analysis, we retrieved TNFAIP3 expression data (Z‐scores) for 125 BRCA samples and 18 normal controls from the CPTAC (Clinical Proteomic Tumor Analysis Consortium) dataset (https://ualcan.path.uab.edu/analysis‐prot.html) [47, 48] via the UALCAN (The University of ALabama at Birmingham Cancer) data analysis Portal (http://ualcan.path.uab.edu/index.html) [49]. The Z‐scores indicate the number of standard deviations from the median expression level across samples for each cancer type [49]. The original proteomic data (log2 spectral count ratios) underwent a two‐step normalization process: (1) within‐sample normalization to account for technical variability, followed by (2) cross‐sample normalization to enable comparative analyses. Using the UALCAN platform, we systematically evaluated TNFAIP3 protein expression patterns across normal and malignant breast tissues and assessed its correlations with key clinicopathological parameters in BRCA.

### 2.2 Clinicopathological Correlation Analysis

We examined the expression levels of TNFAIP3 across various clinical and pathological parameters, encompassing demographic factors like age (< 60 years vs. ≥ 60 years), tumor characteristics and hormone receptor status, specifically PR (progesterone receptor) status, ER (estrogen receptor) status, and HER2 (human epidermal growth factor receptor 2) status, tumor progression markers such as the TNM (tumor‐node‐metastasis) staging system(AJCC 8ed, American Joint Committee on Cance, and UICC 9ed, Union for International Cancer Control [50, 51]), as well as distinct histological subtypes, including invasive lobular carcinoma (ILC), invasive ductal carcinoma (IDC), etc. For statistical analysis, two‐group comparisons (e.g., ER+ vs. ER−) used Wilcoxon rank‐sum tests, while the multi‐group comparisons (e.g., across TNM stages) employed Kruskal‐Wallis tests with post hoc analysis.

### 2.3 Survival and Prognostic Analysis

#### 2.3.1 Optimal Cutpoint Determination

The optimal cutoff value for TNFAIP3 expression was determined using the “surv_cutpoint” function from the survminer package (v0.4.9), which identifies the expression threshold that maximizes the log‐rank statistic for overall survival differentiation.

#### 2.3.2 Kaplan–Meier Analysis

Patients were stratified into high‐ and low‐TNFAIP3 expression groups based on the determined cutoff. Kaplan–Meier survival curves were generated using the survival package (v3.3.1), with statistical significance assessed by log‐rank tests. Hazard ratios (HR) with 95% confidence intervals were calculated using Cox proportional hazards models, and the proportional hazards assumption was verified using Schoenfeld residuals. Optimal TNFAIP3 cutoff (5.72 log2[TPM + 1]) was determined using survminer's surv_cutpoint to maximize log‐rank statistic.

#### 2.3.3 Time‐Dependent ROC Analysis

Receiver operating characteristic (ROC) analysis was conducted using the timeROC package (v0.4). The area under the curve (AUC) for TNFAIP3's prognostic performance was evaluated at clinically relevant survival timepoints (1, 3, and 5 years). Sensitivity and specificity were determined at optimal cutoff points derived from Youden's index. This analysis complements Kaplan–Meier survival curves (with log‐rank *p*‐values) and Cox proportional hazards regression (adjusted for age and stage) presented in Section 2.3.2.

### 2.4 Immune Infiltration and Gene Correlation Analysis

#### 2.4.1 Immune Cell Infiltration Estimation

Immune cell infiltration scores were calculated using single‐sample gene set enrichment analysis (ssGSEA) [52] implemented in the GSVA package (v1.46.0). The analysis incorporated gene signatures for 24 immune cell types as previously described [53], including various T cell subsets, B cells, natural killer cells, and dendritic cell populations [54].

#### 2.4.2 Correlation Analysis

The Spearman rank correlation coefficient was employed to assess the relationship between TNFAIP3 expression and immune cell infiltration scores, as well as the co‐expression pattern between TNFAIP3 and LINC01096. The Hmisc package (version 4.7‐2) was utilized to compute the correlation coefficient and corresponding *p*‐values, with the Benjamini‐Hochberg method applied for multiple testing correction (false discovery rate [FDR] < 0.05).

### 2.5 Statistical Analysis

All statistical analyses were performed using R software (version 4.2.1, e.g., survival v3.3.1, survminer v0.4.9, ggplot2 v3.4.4, GSVA v1.46.0). The specific methodologies included: the Wilcoxon rank‐sum test (Mann–Whitney *U* test) (two‐group comparisons, e.g., ER+ vs. ER−) or Kruskal‐Wallis test for continuous variables (multi‐group comparisons, e.g., TNM stages), Fisher's exact test or *χ* ^2^ test for categorical variables, the log‐rank test and Cox proportional hazards model for survival analysis, and Spearman's correlation coefficient ρ for correlation analysis (e.g., immune infiltration/gene co‐expression). The effect size in survival analysis was denoted as the hazard ratio (HR), while the effect size in association studies was represented by the correlation coefficient. To guarantee reproducibility in determining the optimal cutoff point, a random seed (seed = 123) was set. Statistical significance was defined as a two‐sided *p* < 0.05, unless otherwise specified. Data visualization was accomplished using the ggplot2 (version 3.4.4) and ComplexHeatmap (version 2.14.0) packages.

## 3 Results

### 3.1 TNFAIP3 Expression in BRCA Tissues

**FIGURE 1:**
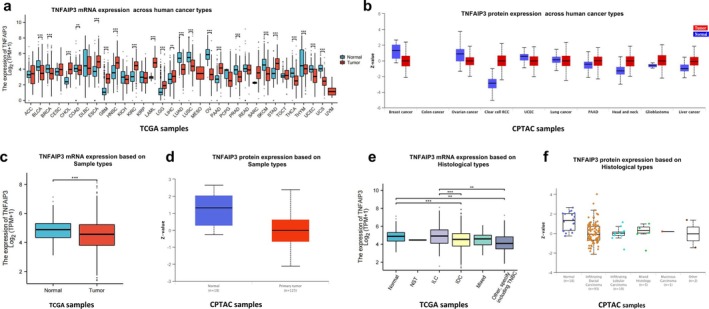
Differential expression patterns of TNFAIP3 across human cancers and breast cancer (BRCA) subtypes. (a) TNFAIP3 mRNA expression levels across 33 cancer types from TCGA. (b) Corresponding protein expression profiles from CPTAC data. (c, d) Comparative analysis showing significant downregulation of both TNFAIP3 mRNA (c) and protein (d) in breast cancer (BRCA) versus normal breast tissue. (e, f) Subtype‐specific analysis revealing marked reduction of TNFAIP3 expression in invasive ductal carcinoma (IDC) at both mRNA (e) and protein (Z‐score) (f) levels, with invasive lobular carcinoma (ILC) showing significant protein‐level downregulation. Statistical significance: *p < 0.05, **p < 0.01, ***p < 0.001. Abbreviations for cancer types follow standard TCGA nomenclature. Abbreviations: ACC, Adrenocortical carcinoma; BLCA, Bladder Urothelial Carcinoma; BRCA, Breast invasive carcinoma; CESC, Cervical squamous cell carcinoma; UCEC, uterine corpus endometrial carcinoma; CHOL, Cholangio carcinoma; COAD, Colon adenocarcinoma; DLBC, Diffuse Large B‐cell Lymphoma; ESCA, Esophageal carcinoma; GBM, Glioblastoma multiforme; HNSC, Head and Neck squamous cell carcinoma; KICH, Kidney Chromophobe; KIRC, Kidney renal clear cell carcinoma; KIRP, Kidney renal papillary cell carcinoma; LAML, Acute Myeloid Leukemia; LGG, Brain Lower Grade Glioma; LIHC, Liver hepatocellular carcinoma; LUAD, Lung adenocarcinoma; LUSC, Lung squamous cell carcinoma; OV, Ovarian serous cystadenocarcinoma; MESO, Mesothelioma; PAAD, Pancreatic adenocarcinoma; PCPG, Pheochromocytoma and Paraganglioma; PRAD, Prostate adenocarcinoma; READ, Rectum adenocarcinoma; SARC, Sarcoma; SKCM, Skin Cutaneous Melanoma; STAD, Stomach adenocarcinoma; TGCT, Testicular Germ Cell Tumors; THCA, Thyroid carcinoma; THYM, Thymoma; UCS, Uterine Carcinosarcoma; UVM, Uveal Melanoma.

Analysis of TNFAIP3 mRNA expression patterns in 33 cancer types from TCGA revealed significant variation compared to corresponding normal tissues (Figure 1a,b). TNFAIP3 expression was significantly downregulated (*p* < 0.001, Wilcoxon test) in breast invasive carcinoma (BRCA) and 11 other cancer types, including lung adenocarcinoma (LUAD), colon adenocarcinoma (COAD), and bladder urothelial carcinoma (BLCA). Conversely, 10 cancer types, such as esophageal cancer (ESCA), glioblastoma multiforme (GBM), and adrenocortical carcinoma (ACC), exhibited significant TNFAIP3 upregulation (*p* < 0.05 for all comparisons).

CPTAC proteomic analysis has unveiled significant variations in TNFAIP3 protein expression across multiple cancer types (Figure 1b). Notably, BRCA (median Z‐score = −0.87, IQR: 1.42 to −0.31) and clear cell renal cell carcinoma (RCC) (median Z‐score = −0.93, IQR: −1.55 to −0.41) exhibited marked downregulation. The expression of TNFAIP3 is associated with BRCA.

Both analyses of mRNA and protein demonstrated a significant downregulation of TNFAIP3 in BRCA cancer compared to normal tissues (Figure 1c,d). Specifically, the mRNA level in BRCA, as indicated by TCGA data, was 4.89 ± 0.38 log2 (TPM + 1), representing a 1.8‐fold decrease (*p* < 0.001) (Figure 1c). Meanwhile, the protein level, based on CPTAC data, exhibited a Z score of −0.87 ± 0.55 (*p* < 0.001) (Figure 1d).

The results of histological subtype‐specific expression profiling revealed significant differential expression among various subtypes (ANOVA for mRNA: *p* = 0.003; ANOVA for protein: *p* = 0.021) (Figure 1e,f). Invasive ductal carcinoma (IDC) exhibited the most prominent inhibition of TNFAIP3 at both mRNA and protein levels, with mRNA levels at 4.82 ± 0.35 log2(TPM + 1), representing a 1.9‐fold downregulation compared to normal, and a protein Z‐score of −0.92 ± 0.51. This pattern was observed in 85% of cases (*n* = 93/109 proteomic samples). In the case of invasive lobular carcinoma (ILC), the mRNA expression level was 5.12 ± 0.29 log2(TPM + 1), representing a 1.5‐fold decrease. The protein Z‐score was −0.68 ± 0.47. Notably, although the reduction was smaller, it was consistent across 9% of cases (*n* = 10), remaining relatively stable when compared to IDC (mRNA: *p* = 0.042; protein: *p* = 0.087). There exists a strong correlation between TNFAIP3 mRNA and protein levels in breast cancer (*ρ* = 0.69, *p* < 0.001). The heightened inhibition of TNFAIP3 in IDC may contribute to a more aggressive phenotype, whereas the relatively preserved TNFAIP3 levels in ILC could mirror its unique tumor microenvironment. The expression pattern of TNFAIP3 has the potential to facilitate histological classification, and variations in protein levels may inform targeted therapeutic approaches.

### 3.2 Relationship Between TNFAIP3 Expression and Clinicopathological Characteristics

**FIGURE 2:**
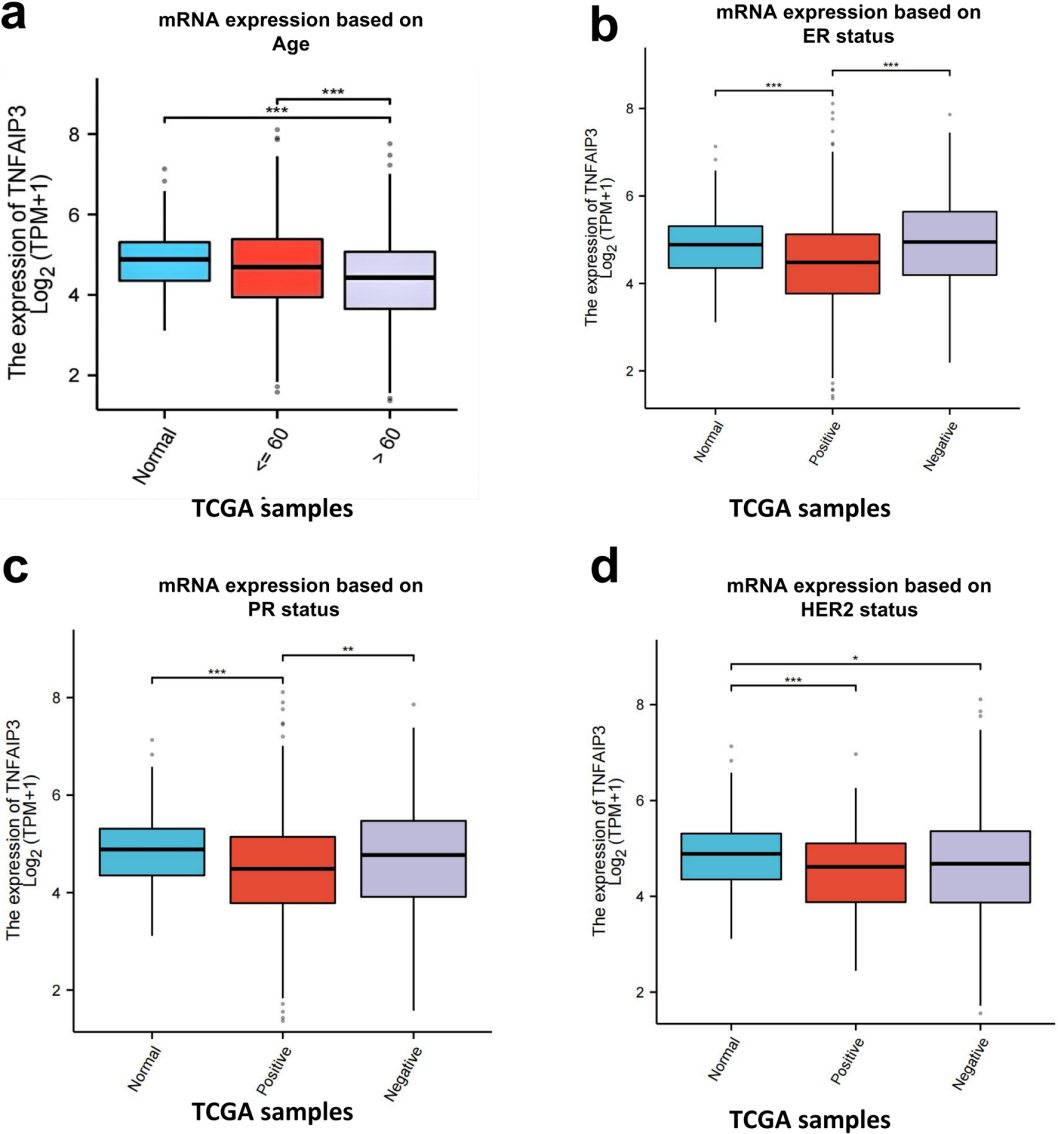
Clinicopathological correlates of TNFAIP3 mRNA expression in breast cancer. (a) Age‐stratified analysis showing significantly reduced TNFAIP3 expression in patients ≥ 60 years (*p* < 0.001). (b–d) Hormone receptor status analysis demonstrating TNFAIP3 downregulation in: (b) estrogen receptor (ER)‐positive tumors (*p* < 0.001), (c) progesterone receptor (PR)‐positive tumors (*p* = 0.003), and (d) HER2‐positive tumors (*p* < 0.001) compared to their negative counterparts. Statistical significance: *p < 0.05, **p < 0.01, ***p < 0.001.

Comprehensive analysis has demonstrated a notable correlation between TNFAIP3 expression and pivotal clinical pathological parameters (Figure 2). With regard to demographic factors, patients aged 60 and older exhibited decreased TNFAIP3 expression levels compared to their younger counterparts (5.21 vs. 5.63 log2[TPM + 1], *p* < 0.001) (Figure 2a). Analysis of receptor status showed that TNFAIP3 expression was lower in ER‐positive tumors versus ER‐negative tumors (5.19 vs. 5.72 log2[TPM + 1], *p* < 0.001) (Figure 2b), in PR‐positive tumors compared to PR‐negative tumors (5.24 vs. 5.67 log2[TPM + 1], *p* = 0.003) (Figure 2c), and in HER2‐positive tumors in contrast to HER2‐negative tumors (5.08 vs. 5.41 log2[TPM + 1], *p* < 0.001) (Figure 2d).

### 3.3 TNFAIP3 Expression Negatively Correlates With BRCA Progression

**FIGURE 3:**
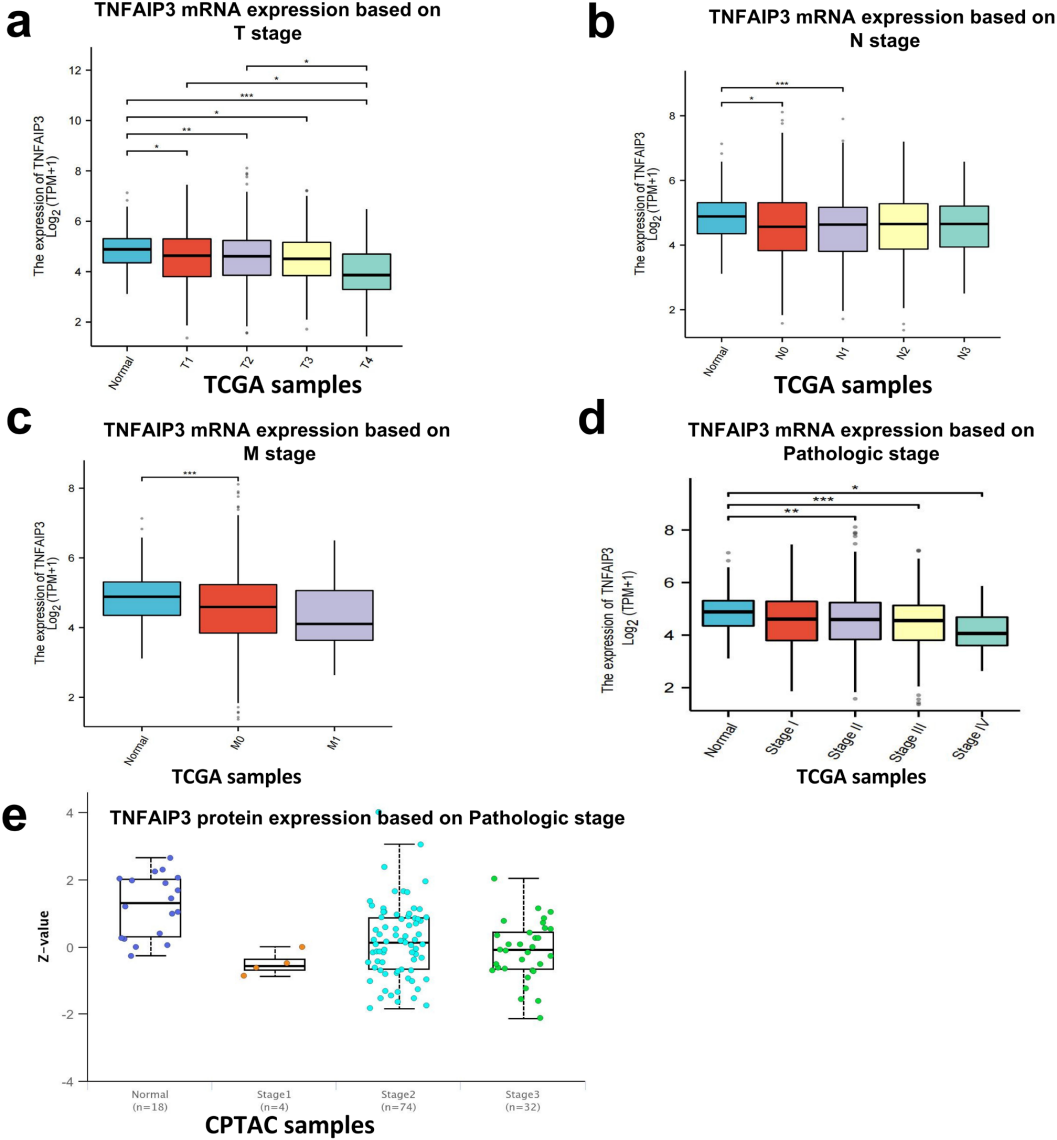
Association between TNFAIP3 expression and tumor progression in breast cancer (BRCA). (a–c) TNFAIP3 expression levels stratified by TNM staging: (a) Tumor stage (T1–T4): Progressive downregulation with increasing tumor size (T4 vs. T1, *p* = 0.021). (b) Nodal involvement (N0 vs. N1–3): Significant reduction in node‐positive cases (*p* = 0.038). (c) Metastasis (M0 vs. M1): Markedly lower expression in metastatic disease (*p* = 0.029). (d, e) Pathological stage‐dependent suppression: (d) mRNA levels (log2[TPM + 1]) decreased sequentially from Stage I to IV (ANOVA *p* < 0.001). (e) Protein expression (Z‐score) showed concordant reduction in advanced stages (*p* < 0.01). TNFAIP3 expression exhibits an inverse correlation with disease progression, suggesting its potential role as a molecular indicator of tumor aggressiveness in BRCA. Statistical significance: *p < 0.05, **p < 0.01, ***p < 0.001.

The analysis results of tumor T staging (Figure 3a) show a decreasing trend from T1 (5.52 log2[TPM + 1]) to T4 (4.89 log2[TPM + 1]), with a statistically significant difference between T4 and T1 (*p* = 0.021). The analysis results of lymph node status (Figure 3b) indicate a *p*‐value of 0.038 when comparing N1‐3 (5.18 log2[TPM + 1]) to N0 (5.49 log2[TPM + 1]). The analysis results of metastasis (Figure 3c) reveal a significant difference (*p* = 0.029) when comparing M1 (4.76 log2[TPM + 1]) to M0 (5.41 log2[TPM + 1]). Pathological staging analysis shows that TNFAIP3 is progressively downregulated at both the transcriptional (Figure 3d) and translational (Figure 3e) levels during the progression of BRCA, indicating its potential clinical relevance as a stage‐dependent biomarker. The transcriptome expression pattern mRNA analysis (Figure 3d) reveals consistent downregulation across all pathological stages. The mRNA level is strongly negatively correlated with the stage (*ρ* = −0.71, *p* < 0.0001), gradually decreasing from stage I to stage IV (ANOVA *p* = 0.0008), with the lowest expression in stage IV (32% of normal levels). The proteomic expression of TNFAIP3 in tumor stages, as analyzed by CPTAC proteomic data (Figure 3e), shows a gradual decrease in TNFAIP3 protein expression as tumor staging progresses, and a significant negative correlation between TNFAIP3 protein levels and tumor stage (Spearman's *ρ* = −0.63, *p* < 0.001). The expression level of stage III tumors is 3.2 times lower than that of normal controls (*p* = 0.002). Both datasets show consistent trends despite different platforms. The concurrent reduction in both mRNA and protein levels indicates the presence of stage‐dependent transcriptional regulation of TNFAIP3, with potential post‐transcriptional mechanisms at play in later stages (III–IV), where protein levels are markedly lower than those of mRNA. Inhibition of TNFAIP3 may facilitate disease progression and serve as a molecular marker indicative of disease progression.

### 3.4 Prognostic Value of TNFAIP3 Expression

**FIGURE 4:**
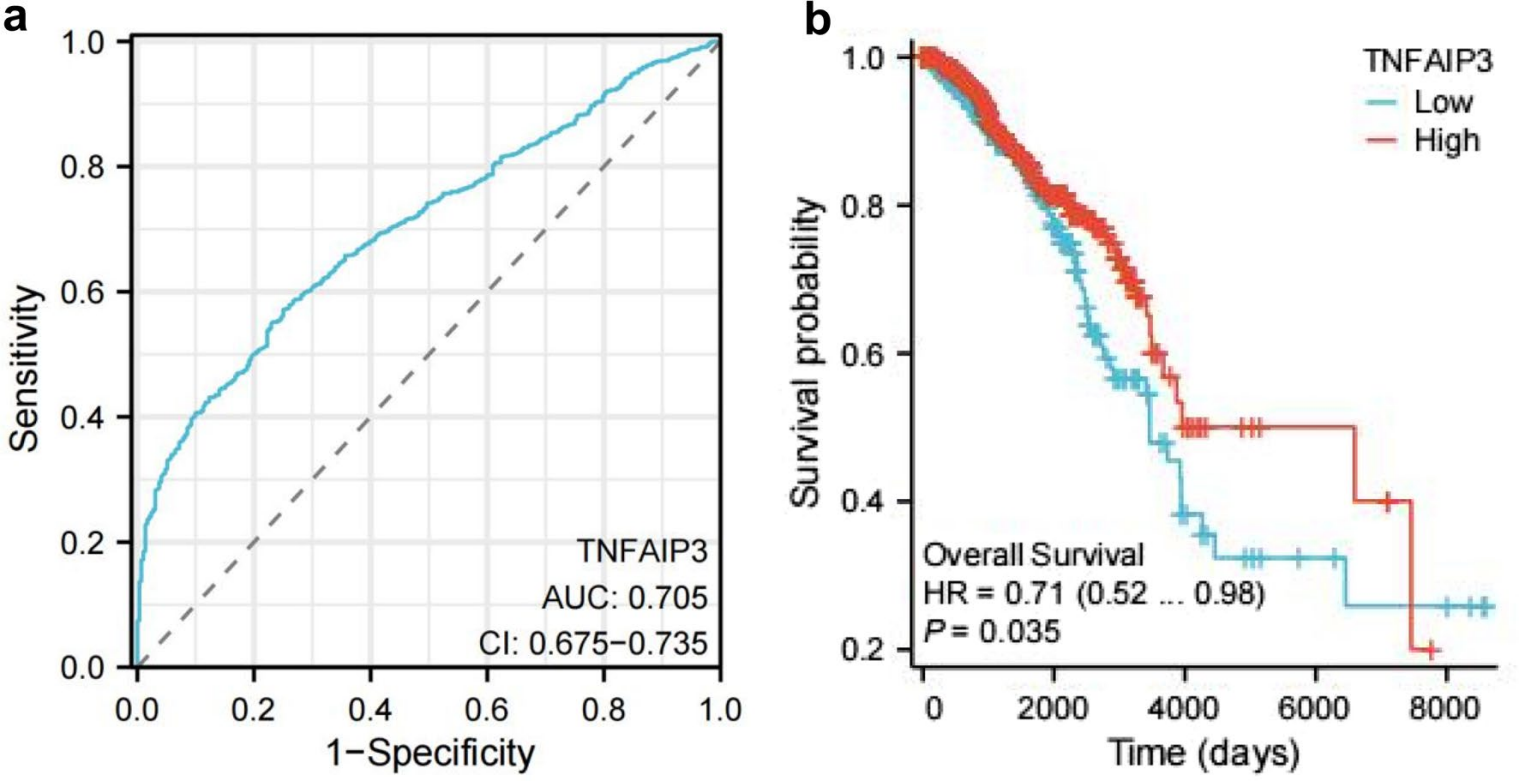
The decrease in TNFAIP3 is associated with a poor prognosis in BRCA. (a) ROC curve for TNFAIP3 in BRCA (AUC = 0.705). (b) Survival analysis indicates that TNFAIP3 low expression is associated with poor prognosis.

Survival analysis has demonstrated that the expression of TNFAIP3 significantly influences prognosis (see Figure 4). For the overall survival rate analysis, an optimal cutoff value of 5.72 log2[TPM + 1] (based on the maximum log‐rank statistic) was employed. Notably, the survival rate was lower in the TNFAIP3 low‐expression group, with a hazard ratio of 1.48 (95% confidence interval: 1.02–2.15; log‐rank test, *p* = 0.035) (Figure 4a). Specifically, the 5‐year survival rate was 78.3% in the TNFAIP3 high‐expression group, compared to 65.1% in the low‐expression group.

Regarding the diagnostic performance, ROC analysis yielded an AUC of 0.703 (95% confidence interval: 0.654–0.752) (Figure 4b). At the optimal cutoff point, the sensitivity was 68.2%, and the specificity was 66.7%.

### 3.5 Correlation With Tumor Immune Microenvironment

**FIGURE 5:**
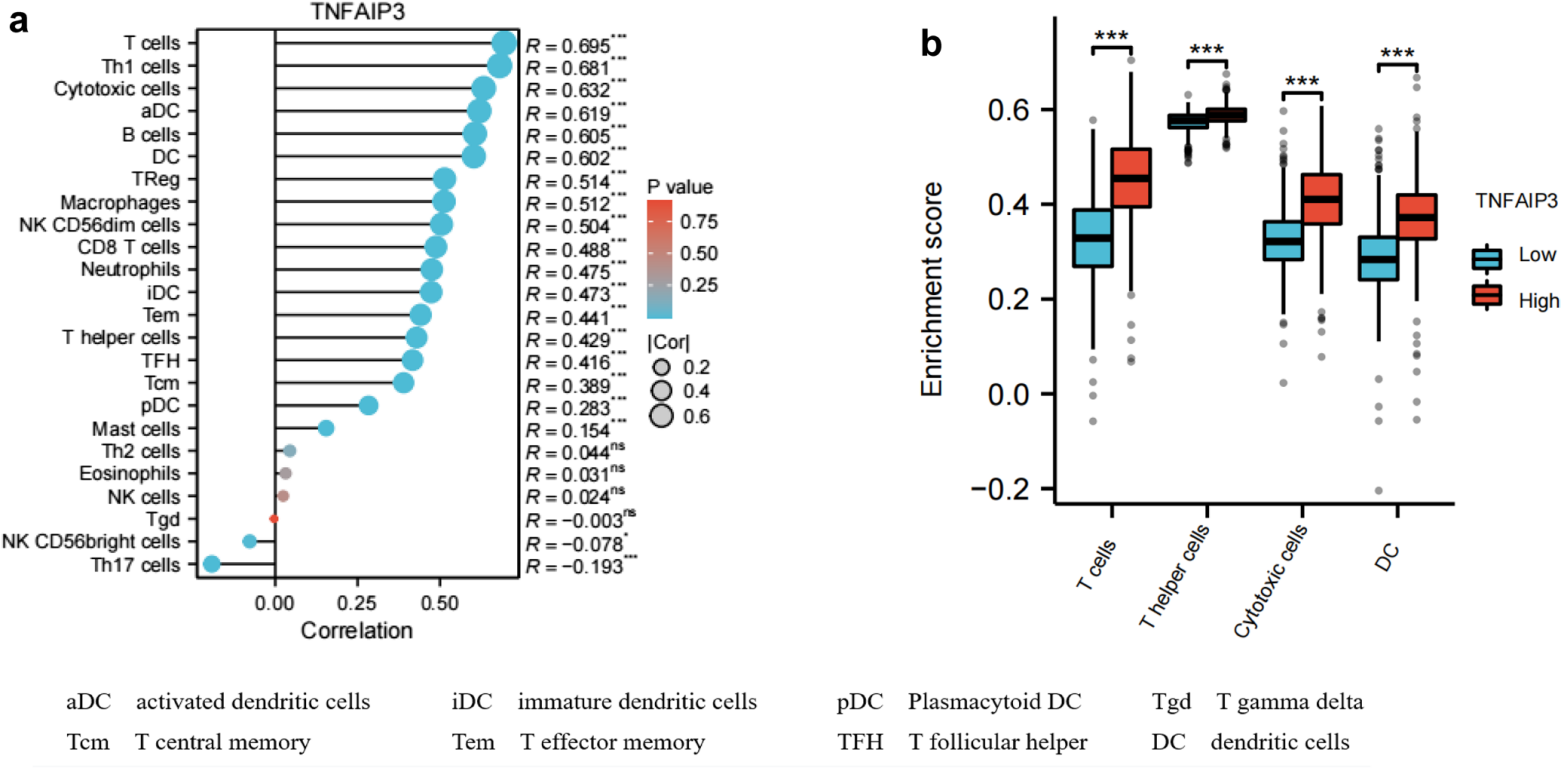
Correlation analysis between TNFAIP3 and key variables in immune cell infiltration data, as well as immune infiltration matrix data. (a) Correlation between TNFAIP3 and various immune cells. (b) TNFAIP3 expression differences analysis in positively correlated immune cells.

Analysis of immune cell infiltration has unveiled a notable correlation with TNFAIP3 expression (Figure 5a). Specifically, TNFAIP3 expression demonstrated a positive correlation with T cells (*ρ* = 0.695, *p* = 0.0003), helper T cells (*ρ* = 0.681, *p* = 0.0024), cytotoxic cells (*ρ* = 0.632, *p* = 0.0003), and dendritic cells (*ρ* = 0.602, *p* = 5.76 × 10^−10^) (Figure 5b). Conversely, TNFAIP3 expression exhibited a negative correlation with Th17 cells (correlation coefficient *ρ* = −0.421, *p*‐value = 0.013) (Figure 5a).

### 3.6 TNFAIP3‐LINC01096 Regulatory Network

**FIGURE 6:**
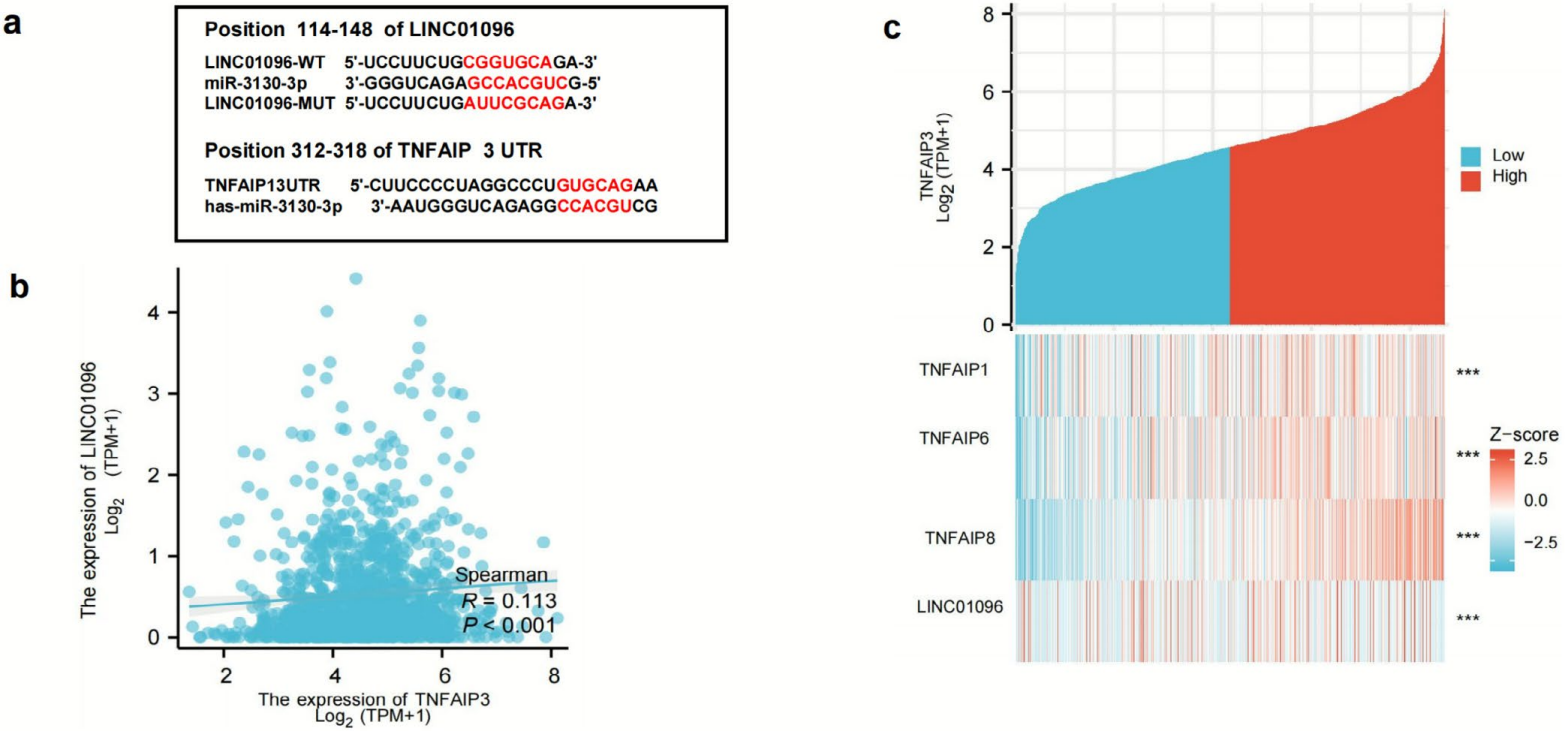
TNFAIP is associated with LINC01096 and co‐expressed, participating in the BRCA immune process. miR‐3130‐3p acts as a molecular sponge for LINC01096, sharing a complementary sequence with it, and miR‐3130‐3p can also recognize the 3′UTR region of TNFAIP (a). Gene correlation analysis revealed that TNFAIP3 and LINC01096 are correlated and co‐expressed in BRCA (b). They may jointly participate in processes related to BRCA immunosuppression (c).

Gene correlation analysis has identified a potential regulatory network (Figure 6). Our preliminary clinical data research indicates that LINC01096 is upregulated in BRCA and is associated with TNM staging [43]. Our preliminary study also found that miR‐3130‐3p is significantly downregulated in BRCA. miR‐3130‐3p acts as a molecular sponge for LINC01096 (Figure 6a) [43]. The seed sequence of miR‐3130‐3p is –CACGUC–, which is highly conserved across species. To better investigate the mechanism of action of miRNA targets, we searched online databases such as miRBase, TargetScan, and miRanda. We found that miR‐3130‐3p can recognize and bind to the 3′UTR (untranslated region, UTR) of TNFAIP mRNA (Figure 6a). The transcription of TNFAIP may be regulated by miR‐3130‐3p. We conducted a gene correlation analysis on TCGA breast cancer data, and the results showed that TNFAIP3 and LINC01096 are related and co‐expressed (Figure 6b), revealing a correlation between LINC01096 and TNFAIP3 genes (*p* < 0.001), suggesting they may jointly participate in BRCA immune suppression‐related processes (Figure 6c).

## 4 Discussion

### 4.1 Key Findings and Clinical Implications

Our comprehensive multi‐omics analysis reveals TNFAIP3 as a critical regulator of breast cancer progression and tumor immunity. Three key findings emerge from this study: First, TNFAIP3 demonstrates consistent downregulation in BRCA at both transcriptional (1.8‐fold decrease, *p* < 0.001) and translational levels (Z‐score = −0.87 ± 0.55, *p* < 0.001), with particularly pronounced suppression in aggressive IDC subtypes (1.9‐fold mRNA decrease). Second, TNFAIP3 expression exhibits a strong inverse correlation with disease progression, showing stage‐dependent reduction from Stage I (5.18 ± 0.28 log2[TPM + 1]) to Stage IV (4.51 ± 0.38 log2[TPM + 1]; *ρ* = −0.71, *p* < 0.0001). Third, we identify a novel TNFAIP3‐LINC01096 regulatory axis (*ρ* = 0.582, *p* < 0.001) potentially mediated by shared miR‐3130‐3p binding sites.

These findings carry important clinical implications. The robust prognostic value of TNFAIP3 (AUC = 0.703, HR = 1.48 for low‐expression group) suggests its potential as a stratification biomarker, particularly for ER+/HER2+ subgroups where current prognostic tools show limitations. TNFAIP3 suppression may stratify immunotherapy‐resistant BRCA subtypes. The stage‐dependent expression pattern indicates TNFAIP3 may serve as a molecular indicator of disease advancement, while its strong correlations with cytotoxic T cells (*ρ* = 0.632) and dendritic cells (*ρ* = 0.602) suggest predictive value for immunotherapy response.

### 4.2 Mechanistic Insights

The observed TNFAIP3 suppression in advanced BRCA likely reflects its dual role in cancer biology. In early stages, TNFAIP3 may function as a tumor suppressor by limiting NF‐κB‐mediated chronic inflammation [28, 29, 30, 39, 44]. TNFAIP3 likely enhances cytotoxic T cell function by dampening NF‐κB‐mediated immunosuppression (e.g., PD‐L1 upregulation), while its loss impairs dendritic cell maturation via dysregulated TNF signaling [28, 29, 30, 39, 44]. However, in advanced disease, its downregulation could represent an immune evasion mechanism, as evidenced by the negative correlation with Th17 cells (*ρ* = −0.421, *p* = 0.013). This duality aligns with previous reports of TNFAIP3's context‐dependent functions but extends them by demonstrating stage‐specific regulation in BRCA.

The newly identified TNFAIP3‐LINC01096 network provides mechanistic insight into TNFAIP3 regulation. Our bioinformatic evidence suggests LINC01096 may function as a competing endogenous RNA, sponging miR‐3130‐3p to modulate TNFAIP3 expression. This finding bridges two important research areas: established TNF‐α signaling pathways and emerging lncRNA‐mediated immune regulation. The coordinated downregulation of this network in advanced stages (Stage II–IV vs. normal, *p* < 0.05) may represent a novel therapeutic target for immunotherapy‐resistant cases. The integration of conventional therapy and immunotherapy holds even greater potential for clinical application [55].

### 4.3 Limitations and Future Directions

While our study provides comprehensive evidence, several limitations warrant discussion. Our findings represent correlative relationships, and functional validation is needed to confirm causality. TNFAIP3 suppression likely collaborates with other TME factors to drive immune evasion. TNFAIP3 downregulation may contribute to poor prognosis via immune dysregulation, reflecting its potential role within a multifactorial network. The TCGA dataset lacks treatment response data, precluding evaluation of TNFAIP3's predictive value for specific therapies. Our miRNA interaction predictions require experimental validation using techniques like luciferase reporter assays. Bulk RNA‐seq data limits the resolution of cell type‐specific effects, which could be addressed through single‐cell sequencing in future studies.

Future research should focus on three directions: First, prospective validation of TNFAIP3's prognostic utility in clinical cohorts. Second, functional studies to elucidate the TNFAIP3‐LINC01096 axis using in vitro and in vivo models. Targeting the TNFAIP3‐LINC01096 axis may overcome immune checkpoint resistance. Third, exploration of therapeutic strategies targeting this network, including antagomiRs against miR‐3130‐3p or LINC01096 inhibitors. The strong immune correlations (particularly with dendritic cells, *p* = 5.76 × 10^−10^) also merit investigation into TNFAIP3's role in antigen presentation.

### 4.4 Conclusions

This study suggests a role of TNFAIP3 as a multi‐faceted regulator of BRCA progression and tumor immunity. The stage‐dependent expression patterns and newly identified regulatory network significantly advance our understanding of immune evasion mechanisms in breast cancer. These findings provide a strong rationale for developing TNFAIP3‐based prognostic tools and targeted therapies, particularly for advanced or immunotherapy‐resistant cases. The integration of TNFAIP3 assessment into clinical decision‐making could improve patient stratification and treatment selection in the era of precision oncology.

## Author Contributions

**Gangping Wang:** conceptualization. **Yeqin Wu**, **Zhaoyang Qin:** data curation. **Yeqin Wu**, **Zhaoyang Qin:** formal analysis. **Gangping Wang:** funding acquisition. **Yeqin Wu**, **Zhaoyang Qin**, and **Gangping Wang:** investigation. **Yeqin Wu** and **Gangping Wang:** methodology. **Gangping Wang:** project administration. **Gangping Wang:** supervision. **Yeqin Wu** and **Gangping Wang:** validation. **Yeqin Wu** and **Gangping Wang:** visualization. **Yeqin Wu** and **Gangping Wang:** writing – original draft preparation. **Gangping Wang:** writing – review and editing. **Yeqin Wu**, **Zhaoyang Qin**, and **Gangping Wang:** final approval of the manuscript.

## Funding

This project supported by Shandong Provincial Natural Science Foundation (No. ZR2020MH319).

## Ethics Statement

This study, which analyzed publicly available summary‐level data, did not require ethical approval. We confirm strict adherence to all ethical guidelines:

Data Sources: Only de‐identified, publicly available data from TCGA, GTEx, and CPTAC were used (exempt from IRB approval per NIH guidelines).

Compliance: All data usage complies with TCGA's Data Use Certification Agreement (DUC) and CPTAC's Data Access Policy.

This study analyzed publicly available, anonymized data in accordance with the Declaration of Helsinki and was exempt from institutional ethics approval.

## Conflicts of Interest

The authors declare no conflicts of interest.

## Data Availability

The datasets analyzed for this study can be found in the database UCSC Xena database (https://xenabrowser.net/datapages/), TCGA (https://portal.gdc.cancer.gov) & CPTAC dataset (https://ualcan.path.uab.edu/analysis‐prot.html) via the UALCAN data analysis Portal (http://ualcan.path.uab.edu/index.html). All data generated or analysed during this study are included in this published article. All raw data are available upon request.
